# Embosphere With or Without Coils in the Treatment of Hemoptysis: A Propensity‐Matched Analysis of Recurrence and Safety

**DOI:** 10.1155/carj/5015609

**Published:** 2026-08-30

**Authors:** Hong-Dou Xu, Liang Yang, Yao Xing, Shi-Bing Hu, Teng Xia

**Affiliations:** ^1^ Department of Interventional Radiology, Gaochun People’s Hospital Affiliated With Jiangsu University, 53 Maoshan Road Gaochun District, Nanjing 210029, Jiangsu, China, jsd.js.cn; ^2^ Department of Thoracic Surgery, Gaochun People’s Hospital Affiliated With Jiangsu University, 53 Maoshan Road Gaochun District, Nanjing 210029, Jiangsu, China

**Keywords:** embosphere, hemoptysis, metallic coils, propensity score matching, subgroup analysis

## Abstract

**Background:**

BAE has become a well‐established, minimally invasive first‐line therapy for hemoptysis. Coils may be useful in selected vascular lesions, including pseudoaneurysms and arteriovenous malformations, but their more proximal occlusive effect may limit distal access and could affect later retreatment and whether adding coils to embosphere embolization improves clinical outcomes remains uncertain. We compared recurrence and safety after embosphere‐based BAE performed with or without adjunctive coils.

**Methods:**

We retrospectively reviewed all consecutive adult patients who underwent first‐time BAE for hemoptysis at our single tertiary care center between January 2019 and December 2024. Patients treated with embosphere were grouped according to whether coils were also deployed. Residual confounding arising from baseline covariate imbalances was mitigated via 1:1 propensity score‐based matching, with matched pairs constrained to a caliper of 0.2 standard deviations of the logit of the propensity score. Kaplan–Meier analysis was used to estimate survival without hemoptysis recurrence, and the results were further analyzed based on the cause of hemoptysis. Factors linked to recurrent hemoptysis were evaluated using Cox proportional hazards models.

**Results:**

The study cohort included 260 patients (median age, 56 years; 161 women), of whom 82 received embosphere plus coils and 178 received embosphere without coils. Propensity score matching yielded 62 patients in each group. At 1, 3, and 5 years prior to matching, recurrence‐free survival stood at 92.6%, 75.0%, and 59.2% in the coil cohort, respectively, compared with 90.9%, 76.0%, and 50.3% in the control cohort (*p* = 0.979). After matching, the corresponding rates were 91.8%, 73.8%, and 51.8% versus 85.2%, 66.8%, and 40.5% (*p* = 0.358). Etiology‐stratified analyses also showed no significant benefit from coil use after matching (bronchiectasis, *p* = 0.770; tuberculosis [TB] sequelae, *p* = 0.868; non‐TB and nonbronchiectasis, *p* = 0.835). The duration of the procedure varied significantly between the groups (*p* = 0.003), while the rates of complications showed no significant difference (*p* = 0.181). On multivariable analysis, TB sequelae emerged as an independent predictor of recurrent bleeding, with a hazard ratio (HR) of 1.69 (95% CI 1.20–2.15, *p* = 0.002). Systemic arterial‐pulmonary shunts (SPSs) were also independently tied to recurrence, carrying an HR of 1.52 (95% CI 1.32–3.72, *p* = 0.048).

**Conclusions:**

In patients undergoing embosphere‐based BAE for hemoptysis, adjunctive coils did not improve long‐term recurrence‐free survival or reduce complications. Coil use was associated with shorter procedure duration.

## 1. Introduction

Hemoptysis is a common respiratory presentation, and approximately 5%–14% of affected patients require urgent intervention [1]. Bronchial artery embolization (BAE) is widely used because it can control bleeding with a minimally invasive approach [2–10]. Despite high rates of initial hemostasis, recurrent hemoptysis remains a clinical problem, with reported recurrence rates of 10%–55% [4–6]. The optimal embolic material has therefore not been settled. A recent study found that N‐butyl‐2‐cyanoacrylate (NBCA) led to longer hemoptysis‐free survival compared to PVA, without raising the risk of complication [8]. However, NBCA requires operator experience because unintended embolization can occur when delivery is poorly controlled.

Gelatin sponge particles are sometimes added at the end of BAE to reinforce occlusion, but their absorbable nature may allow redistribution of flow and embolic material. Metallic coils, which are nonabsorbable, are another adjunct and have been used safely for hemorrhage control in several vascular territories because they are visible and controllable during deployment. A Japanese study reported that coil embolization for BAE had efficacy and safety similar to those of PVA or NBCA [8]. Other investigators have cautioned that coils may close vessels more proximally, which can increase the chance of recurrent hemoptysis and make repeat distal embolization more difficult. For this reason, coils are not routinely selected for all BAE procedures and are often reserved for specific lesions such as pulmonary arteriovenous malformations or aneurysms [9, 10]. In practice, coils may still be combined with particulate agents to strengthen embolization and reduce postembolization washout. Whether adjunctive coils improve outcomes when Embosphere is used for BAE has not been clearly established.

We hypothesized that adding coils to embosphere‐based BAE would not improve long‐term recurrence‐free survival. We compared outcomes when metallic coils were added to embosphere against outcomes with Embosphere only, aiming to guide future choice of embolic agents.

## 2. Materials and Methods

### 2.1. Study Population

**FIGURE 1:**
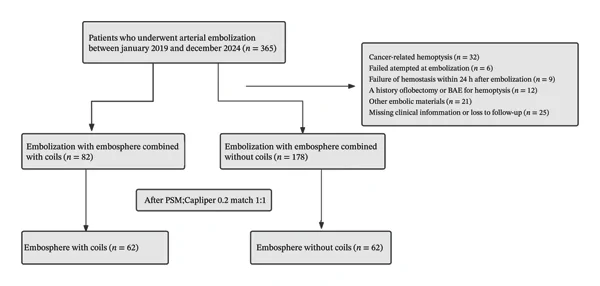
Flowchart of the enrolled patients. Note: PSM, propensity score matching; BAE, bronchial arterial embolization.

We retrospectively reviewed all patients with hemoptysis who underwent arterial embolization at our hospital from January 2019 through December 2024. Eligible patients had confirmed hemoptysis with more than 100 mL of bleeding per episode or inadequate response to standard medical therapy, or persistent blood expectoration that substantially affected quality of life regardless of bleeding volume. Figure 1 summarizes patient selection. Patients were assigned to the coil or noncoil group according to whether coils were used during BAE. Subgroup analyses were performed according to hemoptysis etiology. We obtained contrast‐enhanced computed tomography in all patients prior to embolization, primarily to pinpoint the vessels supplying the active bleeding site.

### 2.2. BAE Procedures

Local anesthesia was achieved with 2% lidocaine at the femoral access site. We used Simmons, RLG, or Cobra catheters to access suspected bleeding feeders. A 2.4–2.8 F Merit Maestro swan‐neck microcatheter was advanced coaxially, positioned as distally as safe near extravasation sites. First‐line embolics were embosphere tris‐acryl microspheres (500–750/700–900 μm, Merit Medical). The attending physician decided whether to use pushable coils or Interlocking Detachable Coils (Boston Scientific Corporation). When coils were used, their diameter was selected to be 10%–20% greater than the culprit artery diameter, allowing for possible vasoconstriction caused by hemostatic drugs. For repeat BAE, embolization was performed proximal to the previously embolized segment if the microcatheter could not traverse existing coils. Newly formed culprit vessels or collaterals were treated using the same approach as in the initial procedure. Technical completion was defined as occlusion of all identified pathological culprit arteries.

### 2.3. Follow‐Up

We graded all complications using the Society of Interventional Radiology (SIR) practice guidelines [10]. Major adverse events were defined as those resulting in extended hospitalization, the requirement for additional interventions, permanent clinical sequelae, or death. After discharge, we checked in with patients via telephone interviews or in‐person outpatient visits, with systematic documentation of recurrent hemoptysis and treatment‐related adverse events. Recurrent bleeding was defined as daily hemoptysis volume ≥ 20 mL, need for repeat BAE, salvage lobectomy, or death attributable to hemorrhage. Time‐to‐recurrence was calculated from the date of successful in‐hospital hemostasis to the date of documented recurrence or final follow‐up.

### 2.4. Statistical Analysis

We report continuous variables as mean ± SD or median (IQR) to report continuous variables. Recurrence‐free survival probabilities were estimated via the Kaplan–Meier procedure. We used 1:1 propensity score matching to iron out baseline differences between the coil group and the noncoil group. Variables that were matched included age, sex, presence of problematic nonbronchial systemic arteries (NBSAs), presence of systemic arterial to pulmonary circulation shunts, underlying lung disease, and the amount of hemoptysis. Patients who died from causes unrelated to hemoptysis were censored at death. Univariate and multivariable Cox proportional hazards models were used to identify predictors of recurrent hemoptysis in the matched cohort. We entered variables with *p* < 0.05 from the univariable analysis into the multivariable model. The analyses were carried out in *R* Version 3.5.3, with *p* < 0.05 being the threshold for statistical significance.

## 3. Results

### 3.1. Clinical Characteristics of Patients With Hemoptysis

Overall, 260 patients with hemoptysis were included. The coil group contained 82 patients, and the noncoil group contained 178 patients. Before matching, the two groups were not well balanced on a few key baseline traits: chronic underlying lung disease including prior tuberculosis (TB) sequelae (*p* < 0.001), how much patients were bleeding at presentation (*p* < 0.001), and systemic arterial‐pulmonary shunts (SPSs, *p* = 0.029). Propensity score matching identified 124 patients, with 62 in each group. After matching, baseline variables were well balanced between groups (Table 1). Bronchial angiographic findings and procedure‐related complications are shown in Table 2.

**TABLE 1:** The study population (*N* = 260).

	Before PSM			After PSM		
Parameters	With coils (*n* = 82)	Without coils (*n* = 178)	*p* value	With coils (*n* = 62)	Without coils(*n* = 62)	*p* value
Age (years), mean ± SD	65.8 ± 8.2	54.6 ± 15.0	0.065	61.5 ± 6.6	61.0 ± 5.8	0.630
Female/Male, No. (%)	55 (67.1)/27 (32.9)	106 (59.6)/72 (40.4)	0.246	37 (59.7)/25 (40.3)	34 (54.8)/18 (45.2)	0.586
Daily hemoptysis volume (mL/d), *n* (%)			0.460			0.766
< 100 mL	15 (18.3)	86 (48.3)	< 0.001	13 (21.0)	15 (24.2)	
100–300 mL	22 (26.8)	52 (29.2)		20 (32.3)	22 (35.5)	
> 300 mL	45 (54.9)	40 (22.5)		29 (46.8)	25 (40.3)	
Underlying lung disease			< 0.001			0.804
Bronchiectasis	28 (34.1)	104 (58.4)		24 (38.7)	25 (40.3)	
TB sequelae	32 (39.0)	28 (15.7)		23 (37.1)	25 (40.3)	
Non‐TB and nonbronchiectasis^∗^	22 (26.8)	46 (25.8)		15 (24.2)	12 (19.4)	
Comorbidities, No. (%)			0.717			0.903
Hypertension	12 (14.6)	30 (16.9)		9 (14.5)	7 (11.3)	
Hepatitis	5 (6.1)	8 (4.5)		3 (4.8)	4 (6.5)	
Cerebral infarction	3 (3.7)	6 (3.4)		2 (3.2)	3 (4.8)	
Diabetes mellitus	10 (12.2)	14 (7.9)		5 (8.1)	4 (6.5)	0.738
Presence of systemic arterial‐pulmonary circulation shunts	52 (63.4)	87 (48.9)	0.029	36 (58.1)	33 (53.2)	0.710
Presence of culprit NBSAs	56 (68.3)	98 (55.1)	0.044	32 (51.6)	30 (48.4)	0.719
Number of culprit bronchial arteries	2.9 ± 0.5	2.4 ± 0.7	0.210	2.2 ± 1.0	2.3 ± 1.0	0.677

Note: Non‐TB and nonbronchiectasis. chronic pneumonia (*n* = 15), pulmonary aspergillosis (*n* = 2), chronic obstructive pulmonary disease (*n* = 7), lung abscess (*n* = 7), and nontuberculous mycobacterial disease (*n* = 7). NBSAs, nonbronchial systemic arteries.

Abbreviation: PSM, propensity score matching.

^∗^includes cryptogenic hemoptysis (*n* = 30).

**TABLE 2:** Details of the procedure and complications.

Parameter	With coils (*n* = 62)	Without coils (*n* = 62)	*p* value
Types of culprit vessels, No. (%)			0.957
Bilateral BA	27 (43.5)	28 (45.2)	
Bilateral BA + others♦	11 (17.7)	9 (14.5)	
Right BA	6 (9.7)	5 (8.1)	
Right BA + others♦	4 (6.5)	2 (3.2)	
Left BA	7 (11.3)	6 (9.7)	
Left BA + others♦	8 (12.9)	10 (16.1)	
Procedure time, (minutes)	93.8 ± 24.0	109.2 ± 29.7	0.003
Complication, No. (%)			0.181
Major	0 (%)	1 (1.6)	1.000
Minor			0.773
Cough	5 (8.1)	7 (11.3)	
Nausea/vomiting	7 (11.3)	6 (9.7)	
Abdominal/chest pain	5 (8.1)	8 (12.9)	
Ecchymosis/hematoma at the puncture site	0 (0)	2 (3.2)	
Allergy	1 (1.6)	4 (6.5)	
Fever	6 (9.7)	5 (8.1)	
Others^∗^	8 (12.9)	9 (14.5)	

*Note:* Others included the inferior phrenic artery, intercostal artery, internal mammary artery, thyrocervical trunk artery or costocervical trunk artery; other complications included back pain (*n* = 8), intramuscular venous thrombosis (*n* = 2), fatigue (*n* = 5), and urinary retention (*n* = 2).

Abbreviation: BA, bronchial artery.

### 3.2. Comparison of the Hemoptysis Recurrence Rate

**FIGURE 2:**
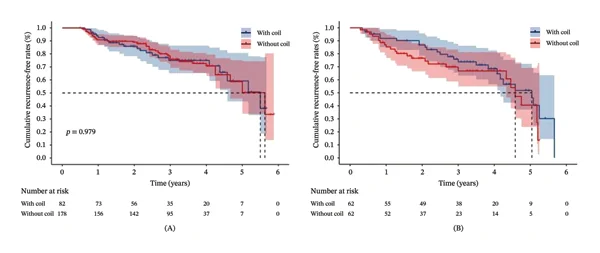
Estimated cumulative hemoptysis recurrence‐free survival curves before (A) and after (B) propensity score matching in patients treated with embosphere with or without coils.

We tracked patients for a median of 34.2 months (IQR 10–62). Before matching, 55 out of 260 patients (21.2%) had at least one recurrence—25 in the coil group and 30 in the noncoil group. At 1, 3, and 5 years, recurrence‐free survival held steady at 92.6%, 75.0%, and 59.2% for those who got coils alongside embosphere. The noncoil group fared similarly, with rates of 90.9%, 76.0%, and 50.3% over the same stretch (Figure 2A). These curves did not differ meaningfully on the log‐rank test (*p* = 0.979). Of the 55 patients with recurrence, 15 (27.3%) underwent repeat BAE, 2 (3.6%) underwent lobectomy, 11 (20.0%) died of pulmonary failure, and 27 (49.1%) received conservative treatment. During repeat BAE, relapse was attributed to missed culprit arteries in 3 patients (1 coil and 2 noncoil), newly formed collateral arteries in 7 patients (4 coil and 3 noncoil), and recanalization in 5 patients (3 coil and 2 noncoil).

Once we balanced the two groups with propensity score matching, 41 out of 124 patients (33.1%) still had recurrent bleeding down the line—23 in the coil‐plus‐embosphere group and 18 in the embosphere‐only cohort. The coil‐plus‐embosphere cohort sustained recurrence‐free survival estimates of 91.8%, 73.8%, and 51.8% at 1, 3, and 5 years, whereas the embosphere‐only cohort showed marginally lower rates of 85.2%, 66.8%, and 40.5% over the same period. Minimal separation was observed between the two survival curves, and log‐rank analysis revealed no clinically or statistically meaningful difference between the treatment arms (*p* = 0.358; Figure 2B).

### 3.3. Subgroup Analysis

**FIGURE 3:**
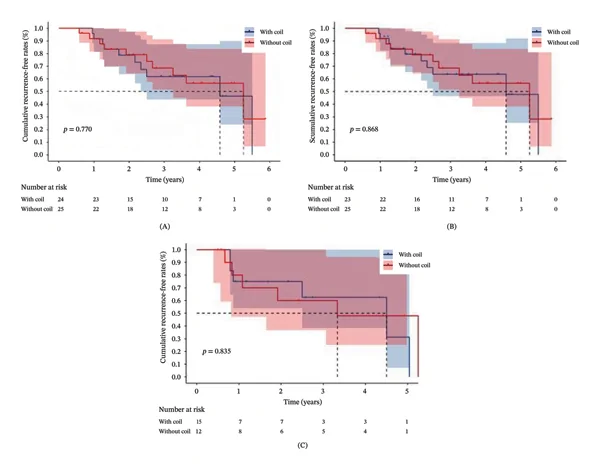
Subgroup analysis of cumulative hemoptysis recurrence‐free survival after propensity score matching by etiology: bronchiectasis (A), TB sequelae (B), and non‐TB and nonbronchiectasis (C).

We next compared recurrence‐free survival across etiologic subgroups after propensity score matching, including bronchiectasis, TB sequelae, and non‐TB and nonbronchiectasis. Coil use was not associated with a significant improvement in any subgroup. For bronchiectasis, the 1‐, 3‐, and 5‐year rates were 83.5%, 70.3%, and 60.3% with coils versus 87.7%, 74.4%, and 63.8% without coils (*p* = 0.770; Figure 3A). For TB sequelae, the corresponding rates were 92.0%, 63.7%, and 47.8% with coils versus 91.8%, 68.5%, and 56.6% without coils (*p* = 0.868; Figure 3B). For non‐TB and nonbronchiectasis, the rates were 75.0%, 62.5%, and 31.3% with coils versus 80.0%, 60.0%, and 48.0% without coils (*p* = 0.835; Figure 3C).

### 3.4. Drivers of Recurrence in the Propensity‐Balanced Cohort

Table 3 shows univariable and multivariable results. After accounting for potential confounders, prior TB sequelae stood out as a strong independent predictor, with a hazard ratio (HR) of 1.69 (95% CI 1.20–2.15, *p* = 0.002). SPSs also independently raised the risk of bleeding relapse, carrying an HR of 1.52 (95% CI 1.32–3.72, *p* = 0.048).

**TABLE 3:** Univariate and multivariable analyses of predictors of recurrent hemoptysis after BAE in the propensity‐matched cohort.

	Univariate		Multivariate	
Variable	HR (95% CI)	*p* value	HR (95% CI)	*p* value
Age (years), mean ± SD	0.98 (0.65–1.21)	0.579		
Sex (male/female)	1.01 (0.84–1.34)	0.952		
Daily hemoptysis volume (mL/d), *n* (%)				
< 100 mL	1.21 (0.52–2.12)	0.671		
100–300 mL	1.27 (0.35–6.45)	0.520		
> 300 mL	1.12 (0.99–1.38)	0.265		
Underlying lung disease				
Bronchiectasis	1.25 (0.97–1.67)	0.062		
TB sequelae	2.54 (2.11–3.34)	0.012	1.69 (1.20–2.15)	0.002
Non‐TB and nonbronchiectasis	1.94 (0.89–3.21)	0.524		
Comorbidities, No. (%)				
Hypertension	1.35 (0.69–2.45)	0.427		
Hepatitis	1.20 (0.98–1.02)	0.845		
Cerebral infarction	1.33 (0.53–2.54)	0.964		
Diabetes mellitus	1.51 (0.93–1.21)	0.421		
Presence of systemic arterial‐pulmonary circulation shunts	2.62 (1.45–3.27)	< 0.001	1.52 (1.32–3.72)	0.048
Presence of culprit NBSAs	1.18 (0.92–1.43)	0.162		
Number of culprit bronchial arteries	1.15 (0.89–1.28)	0.081		

*Note:* NBSAs, nonbronchial systemic arteries.

### 3.5. Comparison of Complications Associated With Interventional Procedures

**FIGURE 4:**
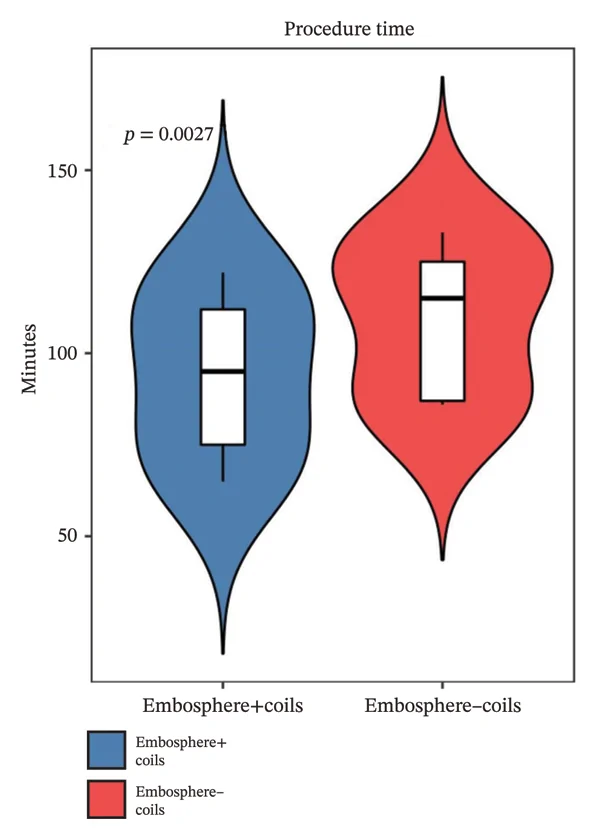
Comparison of procedure time between the embosphere with coils group and embosphere without coils g.

Compared with embosphere embolization without coils, embosphere embolization with adjunctive coils was associated with a shorter procedure time (93.8 ± 24.0 min vs. 109.2 ± 29.7 min; *p* = 0.003; Figure 4). Postmatching complication rates were 51.6% (32/62) with coil + embosphere vs. 67.7% (42/62) with embosphere alone (*p* = 0.181; Table 2). Only one major complication occurred in the embosphere‐only group: an elderly patient had an iatrogenic aortic dissection during left bronchial artery catheterization. It was managed conservatively, and the patient stayed stable without symptoms.

## 4. Discussion

This retrospective study assessed the efficacy and safety of embosphere‐based BAE for hemoptysis with and without adjunctive coils. Adding coils did not improve recurrence‐free survival compared with embosphere embolization alone. Coil use was associated with shorter procedure duration, but complication rates were not significantly different. TB sequelae and SPSs were independently associated with a higher risk of recurrent hemoptysis.

The main purpose of this analysis was to determine whether metallic coils add clinical benefit when used with embosphere during BAE. After matching, the coil‐plus‐embosphere group held onto 1‐, 3‐, and 5‐year hemoptysis recurrence‐free survival rates of 91.8%, 73.8%, and 51.8% over the full follow‐up window. The embosphere‐only cohort lagged just slightly behind, with corresponding rates of 85.2%, 66.8%, and 40.5%. Subgroup analyses showed the same general pattern across etiologies. We therefore found no evidence that adjunctive coils improved recurrence control or safety. Outcomes in our cohort were broadly consistent with earlier reports, although differences in underlying disease, embolization technique, and embolic level may explain variation across studies. Yan et al. [11] reported 1‐, 3‐, and 5‐year recurrence‐free rates of 88.0%, 76.2%, and 73.8% after BAE. In our study, complications were not significantly different between the coil and noncoil groups (51.6% vs. 67.7%; *p* = 0.181), similar to previous reports [12, 13]. The only major complication was aortic dissection in the noncoil group, probably related to aortic wall vulnerability during catheter manipulation. Spinal artery ischemia after BAE is serious but uncommon, with reported rates below 1% [12–14]. Coil‐specific adverse events have also been described. According to Ishikawa et al. [15], there were two cases where coils unpredictably migrated from the bronchial artery into the bronchus after BAE. Several pathophysiologic pathways likely contribute to this observation. Ischemic necrosis of the bronchial wall represents the most commonly cited driver, particularly when the target bronchial artery lies in direct apposition to the submucosal layer. The inherent structural vulnerability of the membranous bronchus further exacerbates this risk, compounded by chronic pulsatile stress transmitted from the bronchial artery through the metallic coil construct.

Several embolic agents are available for BAE, and each has practical advantages and limitations. In many procedures, more than one material is used to obtain durable and controlled occlusion. PVA and microsphere particles are commonly selected [7, 13, 16–18], and a recent comparison reported similar efficacy and safety for microspheres and PVA in patients with hemoptysis [19]. Gelatin sponge particles may be used after other agents to complete embolization, but they can be absorbed over a short period and are therefore not preferred as the sole embolic material. In patients treated with gelatin sponge for pulmonary aspergilloma, recurrence at 1 year was reported in 62% of those with aspergillosis and 28% of those with other causes of hemoptysis [20]. From a mechanistic perspective, durable distal and selective embolization with permanent agents should reduce recurrence. The combination of NBCA and iodized oil can be more effective in controlling hemoptysis than PVA [7], although it may elevate the risk of cerebral or systemic embolism when used by operators lacking experience. The role of coils remains debated. Some authors suggest that coil embolization may prevent recurrence related to proximal embolization [21], whereas others emphasize that coils are not blood flow‐dependent and may be useful with newer designs, including high‐packing‐density, hydrogel‐polymer‐coated, or highly thrombogenic coils that can preserve spinal supply, protect distal circulation, or occlude large bronchial‐to‐pulmonary shunts [5]. Ishikawa et al. [8] found that detachable coils achieved safety and long‐term efficacy comparable to PVA or NBCA. One explanation is that recurrent bleeding can still be treated by embolizing immediately proximal to a previous coil site when a microcatheter is used. Microcatheter use during the first embolization may also reduce recurrence risk. After BAE. When hemoptysis recurred after the initial BAE, we traced three overlapping drivers in our cohort—none of which pointed to a clear benefit of adding coils. First, technical slip‐ups: we occasionally missed a tiny feeder, left a target artery partially occluded, or watched a treated vessel reopen as collaterals kicked in to compensate. Second, underlying lung disease progression: even a perfect initial embolization cannot stop TB sequelae or chronic inflammation from driving new vessel formation and bleeding over time. Third, the role of coil recanalization—where we diverged sharply from prior reports. Ryuge et al. found coil reopening was the dominant recurrence driver in their series [22], but only 3 of the 8 repeat procedures in our coil group were attributed to recanalization (37%), far lower than their 62% rate. We think this gap reflects our routine use of microcatheters for deep distal embolization before coil deployment—a step that may reduce the risk of proximal coil shift and late reopening. Our data also confirmed two well‐known risk amplifiers: TB sequelae and SPSs. Years of TB‐driven parenchymal destruction fuel bronchial artery hypertrophy and abnormal arteriovenous communications, creating a substrate for recurrent bleeding even after technically successful embolization [23]. SPSs add another layer of complexity: these abnormal systemic‐pulmonary connections are prone to remodeling and recanalization, making long‐term hemostasis harder to sustain regardless of the embolic agent used.

There are limitations to this study. Primarily, the sample size was limited, and multicenter cohorts are required to verify these findings. Second, prognosis may be affected by treatment of the underlying respiratory disease, including antibiotic therapy, oxygenation status, and inflammatory burden. Third, this was a single‐center retrospective observational study, and residual selection bias may remain despite propensity score matching. The decision to use coils depended on operator judgment and experience, which may have influenced embolic agent selection. Coils may still be preferable in selected settings, such as aneurysm‐ or arteriovenous malformation‐related hemoptysis.

In conclusion, adjunctive coils did not improve the efficacy or safety of embosphere‐based BAE for hemoptysis compared with embosphere embolization without coils. Metallic coils seemed to streamline the embolization process, consistently leading to procedure times compared to embosphere‐only cases [24].

## Author Contributions

Hong‐Dou Xu conceived and designed the study. Shi‐Bing Hu collected the clinical data. Hong‐Dou Xu and Liang Yang analyzed the data and drafted the manuscript. Teng Xia revised the manuscript and approved the final version.

## Funding

The authors have nothing to report.

## Ethics Statement

The Ethics Committee of Nanjing Gaochun People’s Hospital waived the requirement for informed consent because of the retrospective study design.

## Conflicts of Interest

The authors declare no conflicts of interest.

## Data Availability

Raw data and code are available from the corresponding author upon reasonable request.
